# Comprehensive Genomic Profiling Across Diverse Solid Tumors: A Real-World Experience From India With FoundationOne®CDx Testing

**DOI:** 10.7759/cureus.101804

**Published:** 2026-01-18

**Authors:** Pramod K Julka, Deepika Arya, Sanjay Gupta

**Affiliations:** 1 Medical Oncology, Max Super Speciality Hospital, Saket, Delhi, IND; 2 Clinical Research, Catalyst Clinical Services Pvt. Ltd., Delhi, IND

**Keywords:** foundationone®cdx, genomic profiling, ngs testing, precision oncology, somatic mutations

## Abstract

Introduction

Precision oncology relies on tumor-specific genomic profiling, and broad next-generation sequencing (NGS) assays such as FoundationOne®CDx (F1CDx) are increasingly used to guide targeted therapy. This study aimed to evaluate the mutational landscape, actionable alterations, and clinical utility of F1CDx testing across diverse solid tumors at an Indian oncology center.

Materials and methods

This retrospective study included adult patients (≥18 years) who underwent comprehensive genomic profiling with the F1CDx assay between August 2017 and April 2025. De-identified demographic, clinical, and genomic data were extracted from medical records. Somatic alterations were prioritized using a predefined hierarchy, and gene-level alteration frequencies were calculated using a binary mutation matrix. Descriptive statistics were employed to summarize all clinical and molecular findings. Genomic actionability in this study primarily reflects report-level or biological actionability, with only a subset translating into guideline-endorsed and accessible therapies in Indian practice.

Results

A total of 115 patients received NGS testing (67.8% female; median age was 57 years). Breast cancers (n=35), gastrointestinal malignancies (n=22), thoracic or lung (n=18), and gynecologic cancers (n=15) were most common. Distinct tissue-specific genomic signatures were observed, with recurrent convergence on MAPK, PI3K/AKT/mTOR, RTK, DNA repair, and cell cycle pathways. Breast cancers demonstrated TP53 mutations, PI3K/AKT activation, and recurrent 8p11-12/11q13 amplifications, with infrequent findings such as IDH1 R132C. Gastrointestinal cancers were dominated by KRAS/NRAS and WNT-pathway alterations, with minimal evidence of mismatch repair (MMR) deficiency. Lung cancers showed typical non-small cell lung cancer (NSCLC) driver alterations (KRAS G12D, RET fusions, PIK3CA mutations) and pervasive tumor suppressor loss, with no microsatellite instability (MSI)-high cases. Gynecologic cancers showed strong PI3K-pathway activation, TP53 disruption, and multiple amplification-driven oncogenic programs. Less frequent tumor types, including prostate, hepatobiliary, glioblastoma (GBM), head and neck, urologic cancers, and sarcomas, exhibited established genomic drivers. F1CDx recommended FDA-approved therapies relevant to the tumor type in 41.7% of cases and tumor-agnostic or off-label therapies in 50.4%.

Conclusions

F1CDx profiling identified a broad spectrum of actionable alterations across diverse tumors, highlighting its strong potential to expand therapeutic options and advance precision oncology in real-world Indian practice.

## Introduction

Precision medicine, also known as targeted medicine, genomic medicine, or personalized medicine, has become a transformative approach in modern oncology [1]. Although only 8-9% of cancer patients in the United States were eligible for genomically driven therapies in 2018, the outcomes among treated individuals were markedly superior to traditional chemotherapy, with a median response rate of 54% and a median response duration of 30 months [2]. Nevertheless, limited access to targeted drugs and restricted availability of molecular diagnostic testing continue to impede widespread implementation of precision oncology [3]. The use of broad multigene next-generation sequencing (NGS) tumor panels has enhanced the detection of actionable genomic alterations, expanded treatment options, and advanced precision cancer care [4-5]. Although integrating large-scale genomic data into routine practice adds complexity, the rapidly growing body of molecular knowledge continues to move oncology closer to its goal of delivering the right treatment at the right dose and time with minimal toxicity and maximal benefit [6].

NGS remains central to precision oncology, offering simultaneous sequencing of millions of DNA fragments and providing detailed insights into genome structure, genetic variants, gene expression, and functional alterations [7]. It also quantifies tumor mutational burden (TMB) and detects hypermutated signatures linked to DNA repair defects and potential immunotherapy response [6]. NGS is especially valuable in advanced cancers and offers important therapeutic options for rare malignancies [6]. Several NGS-based profiling platforms now exist, each assessing distinct gene sets [8]. Among these, FoundationOne®CDx (F1CDx), approved by the U.S. FDA in 2017 as the first broad NGS panel for all solid tumors [5, 9-10], is one of the most widely adopted and is extensively used in clinical research and drug development [11-12]. Other FDA-cleared NGS diagnostics include MSK-IMPACT, FoundationFocus CDx BRCA LOH, the Oncomine Dx Target Test, and the Illumina Extended RAS panel [6].

F1CDx evaluates 324 cancer-associated genes, including coding exons of 309 genes, one promoter region, one noncoding RNA, and selected intronic regions of 34 commonly rearranged genes, 21 of which are also assessed at the coding level [13]. It simultaneously reports microsatellite instability (MSI) and TMB, enabling a comprehensive molecular profile [14-15]. Studies show that Foundation Medicine® testing identifies more mutations and potential therapeutic targets than on-site assays [3]. Alterations are classified as known or likely pathogenic variants or as variants of unknown significance (VUS) [16]. The assay has a high technical success rate of 89.1%, even in specimens up to 876 weeks old [5], with similar performance reported internationally [3,17-18].

Taken together, while broad NGS-based tumor profiling has transformed the identification of actionable genomic alterations and expanded therapeutic possibilities, real-world evidence on its clinical yield and practical utility remains limited, particularly in low- and middle-income countries where access, cost, and guideline alignment pose additional challenges. Most available data are derived from clinical trials or Western populations and may not reflect routine practice or population-specific genomic landscapes. In this context, the present study evaluates the real-world application of comprehensive genomic profiling using F1CDx testing across diverse solid tumors in an Indian oncology setting: to characterize the spectrum of detected alterations, estimate report-level actionability, and assess its pragmatic relevance to clinical decision-making in India.

## Materials and methods

Study design and setting

This retrospective, real-world study was conducted at a high-volume oncology daycare center in the Indian subcontinent. Medical records of adult patients (≥18 years) who underwent NGS using the F1CDx assay between August 2017 and April 2025 were reviewed. Only patients with complete and verifiable documentation of all required clinical and demographic variables were included in the final analysis. The study was conducted in accordance with the principles of the Declaration of Helsinki and adhered to Good Clinical Practice (GCP) guidelines. Ethical approval was granted by the Institutional Ethics Committee vide letter BHR/RS/MSSH/DDF/SKT-2/IEC/ONCO/25-32.

Data collection

De-identified patient data were extracted from the institutional medical record system using a standardized, predesigned proforma. Variables collected included baseline demographics, relevant medical history, disease management group (DMG), and detailed outcomes of NGS testing. Genomic data were extracted from the F1CDx reports and compiled into a structured worksheet for patient-level analysis of somatic alterations. All mutation entries were systematically parsed to isolate the gene symbol and corresponding variant. When multiple alterations were reported for the same gene in a single patient, only the highest-impact event was retained according to a predefined hierarchy (amplification, loss/deletion, frameshift, nonsense, splice-site, missense, and other). The hierarchical approach was used to enhance clarity in summary tables and pathway maps; however, this may under-represent clinically relevant co-alterations, including resistance mechanisms or dual hits, which are better captured at the individual patient level.

A binary alteration matrix was subsequently generated, with genes represented as rows and patients as columns, indicating the presence or absence of an alteration in each individual. A gene was classified as altered if at least one prioritized variant was detected for that patient. Patient counts, therefore, reflected the number of individuals harboring an alteration in each gene, and alteration frequencies were calculated as the proportion of the total cohort, rounded to one decimal place. This standardized workflow ensured consistent representation of biologically meaningful somatic events and enabled reliable comparative visualization and downstream genomic analyses. The actionability in this study primarily reflects report-level or biological therapeutic suggestions, of which only a subset translate into guideline-endorsed and accessible treatments in Indian clinical practice. All personally identifiable information was removed before data entry and analysis to ensure confidentiality.

Statistical analysis

Categorical variables were summarized using frequencies and percentages, while continuous variables were expressed as means with standard deviations (SD) or medians with ranges, as appropriate. All statistical analyses were performed using Stata version 16.0 (StataCorp LLC, College Station, TX).

## Results

Between August 2017 and April 2025, medical records of 115 patients who underwent comprehensive genomic profiling using the F1CDx NGS platform were identified and reviewed. The cohort comprised 78 females (67.8%) and 37 males (32.2%), with a median age of 57 years (range: 28-80 years). The most common DMG was breast cancer (n=35), followed by gastrointestinal malignancies (n=22), thoracic/lung cancers (n=18), and gynecologic cancers (n=15). Less frequent DMG included head and neck cancers (n=8), GBM/primary brain tumors (n=5), prostate cancer (n=4), urologic cancers (n=3), liver cancers (n=3), and sarcoma/musculoskeletal tumors (n=2). The demographic characteristics and DMG of patients who underwent NGS testing are summarized in Table 1.

**Table 1:** Baseline characteristics of the study population (N=115)

Variables	Values
Age, years, median (range)	57 (28-80)
Gender, n (%)	
Male	37 (32.2)
Female	78 (67.8)
Disease Management Group, n (%)	
Breast cancer	35 (30.4)
Gastrointestinal malignancy	22 (19.1)
Lung cancer	18 (15.7)
Gynecologic cancer	15 (13.0)
Head and neck cancer	8 (7.0)
Glioblastoma multiforme (GBM)	5 (4.3)
Prostate cancer	4 (3.5)
Urologic cancer	3 (2.6)
Liver cancer	3 (2.6)
Sarcoma	2 (1.7)

Comprehensive genomic profiling across the full multi-tumor cohort revealed distinct, tissue-specific molecular signatures alongside several convergent oncogenic pathways. In the breast cancer cohort (n=35), tumors demonstrated low-intermediate TMB and were largely microsatellite stable (MSS). The genomic landscape was dominated by TP53 mutations (62.9%), PI3K/AKT pathway activation (PIK3CA ~37%; PTEN ~14%), and recurrent 8p11-12/11q13 amplifications involving FGFR1, NSD3, ZNF703, CCND1, and MYC. Additional alterations included ERBB2 events (8.6%), CDH1 mutations (8.6%), and homologous recombination deficiency (HRD)-associated genes such as BRCA1 and RAD21 (Table 2).

**Table 2:** Clinically relevant mutations: breast cancer cohort (N=35)

Gene	Pathway	N (%)	Clinical relevance
Tier 1 - Directly targetable			
PIK3CA	PI3K/AKT/mTOR	13 (37.1)	Predictive for PI3K inhibitors (alpelisib)
ERBB2	HER2/RTK	3 (8.6)	HER2-targeted therapies
BRCA1	HRD/DNA repair	2 (5.7)	PARP inhibitor sensitive; hereditary
ESR1	Hormone receptor	2 (5.7)	Endocrine resistance; SERD-responsive
FGFR2	FGFR/RTK	2 (5.7)	FGFR-targeted trials
BRCA2	HRD/DNA repair	1 (2.9)	PARP inhibitor sensitive; hereditary
NTRK1	TRK/RTK	1 (2.9)	TRK fusion–targeted therapy if fused
ROS1	RTK	1 (2.9)	Fusion-targeted therapy if rearranged
Tier 2 - Prognostic/resistance			
TP53	Tumor suppressor	22 (62.9)	Poor prognosis; genomic instability
PTEN	PI3K/AKT/mTOR	5 (14.3)	Endocrine resistance; pathway activation
RB1	Cell cycle	3 (8.6)	Predicts CDK4/6 inhibitor resistance
CDH1	Adhesion/lobular	3 (8.6)	Lobular CA phenotype
Tier 3 - Actionable amplifications			
MYC	Amplification pathway	8 (22.9)	High proliferation; poor prognosis
CCND1	Amplification pathway	6 (17.1)	Supports CDK4/6 inhibitor use
FGFR1	Amplification pathway	4 (11.4)	Endocrine resistance; FGFR trials
NSD3	Amplification pathway	4 (11.4)	8p11 amplicon; aggressive
ZNF703	Amplification pathway	4 (11.4)	Luminal B; adverse
MCL1	Amplification pathway	3 (8.6)	Anti-apoptotic
ZNF217	Amplification pathway	2 (5.7)	Luminal B; adverse
MDM4	Amplification pathway	2 (5.7)	TP53 suppression
MDM2	Amplification pathway	1 (2.9)	TP53 suppression; trials
Tier 4 - HRD-associated			
RAD21	HRD/DNA repair	3 (8.6)	HRD; cohesion complex
CHEK2	HRD/DNA repair	1 (2.9)	HRD; PARP-sensitive potential
BARD1	HRD/DNA repair	1 (2.9)	HRD; hereditary
BRIP1	HRD/DNA repair	1 (2.9)	HRD; hereditary
FANCA	HRD/DNA repair	1 (2.9)	Fanconi; HRD
Tier 5 - Pathway modifiers			
MAP3K1	MAPK/RAS	2 (5.7)	Luminal endocrine response
KRAS	MAPK/RAS	2 (5.7)	MAPK activation
MAP2K4	MAPK/RAS	1 (2.9)	Endocrine resistance
NF1	MAPK/RAS	1 (2.9)	RAS pathway activation
Tier 6 - Epigenetic/chromatin			
NSD3	Chromatin	4 (11.4)	Chromatin/8p11
ASXL1	Chromatin	2 (5.7)	Epigenetic deregulation
CREBBP	Chromatin	2 (5.7)	Transcription co-activator
KDM5A	Chromatin	1 (2.9)	Epigenetic reprogramming
SMARCB1	Chromatin	1 (2.9)	SWI/SNF complex
Tier 7 - Other/low Evidence			
EMSY	BRCA regulator	1 (2.9)	Phenocopies BRCA1 loss
CD274	Immune checkpoint	1 (2.9)	PD-L1 amplification
PDCD1LG2	Immune checkpoint	1 (2.9)	PD-L2 amplification

**Figure 1:**
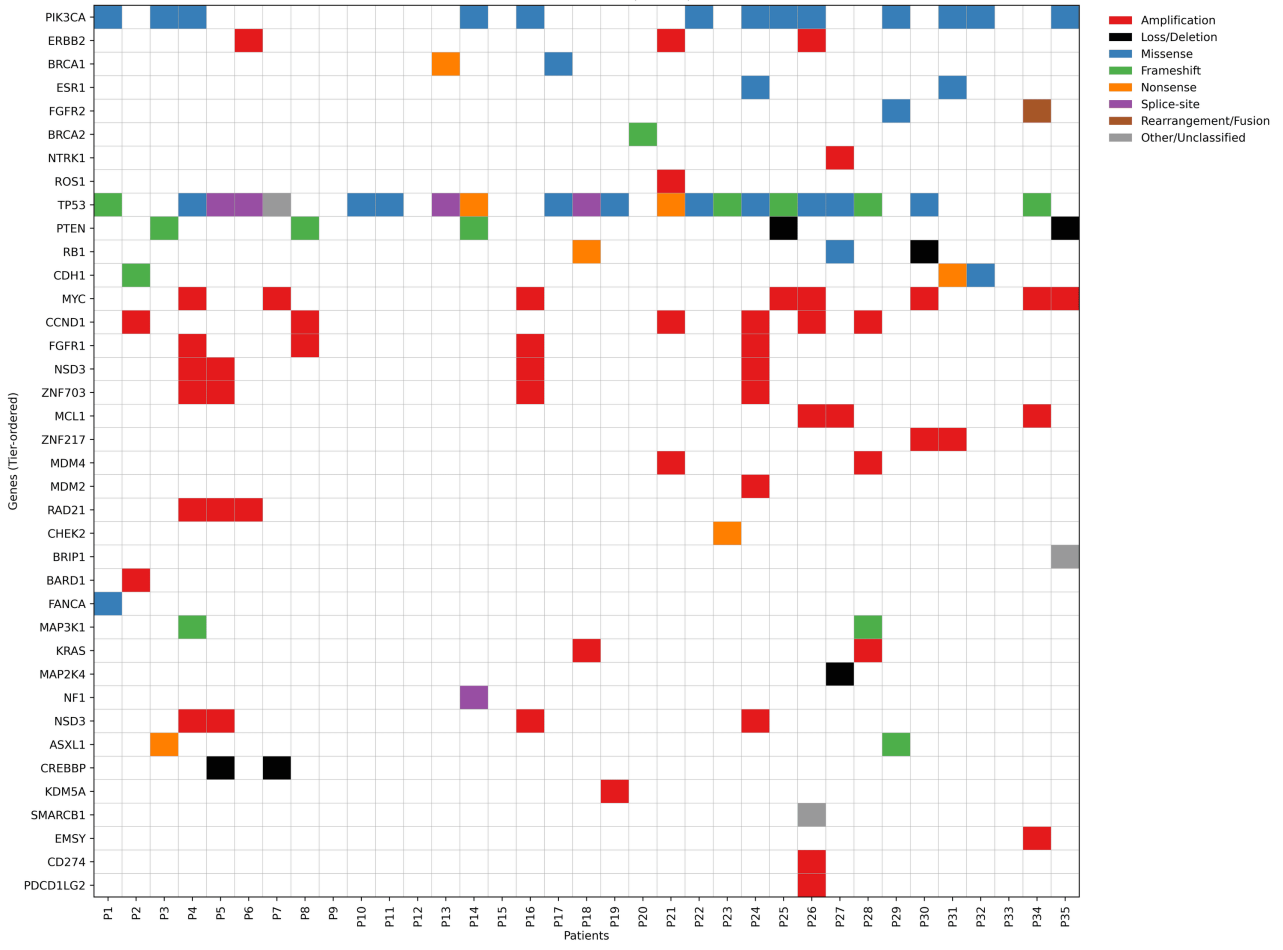
Somatic mutation map of breast cancer patients by key oncogenic pathways (N=35)

Many of the observed amplifications, particularly involving 8p11-12/11q13, correspond to trial-level or exploratory molecular signals rather than established routine therapeutic targets. Together, these features reflect enrichment of aggressive luminal B and HER2-enriched subtypes with multiple actionable nodes. In this cohort, one clinically significant observation was identified. One patient presented with an IDH1 R132C mutation, an alteration considered an off-label therapeutic target in breast cancer, as IDH1 inhibitors are currently approved only for acute myeloid leukemia, cholangiocarcinoma, and select central nervous system tumors. The detection of this mutation in the cohort underscores a rare but potentially relevant molecular event that merits individualized clinical interpretation. Figure 1 illustrates the somatic mutation map of breast cancer patients across key oncogenic pathways.

The gastrointestinal cancer cohort (n=22) showed a mutation profile driven primarily by MAPK-pathway activation (KRAS, NRAS) and WNT-pathway dysregulation (APC, CTNNB1), accompanied by widespread TP53 and SMAD4 loss. PI3K-pathway events (PIK3CA, PIK3R1, PTEN) were also present. HRD-related mutations (BRCA1/2, ATM, BRIP1) occurred in a subset, whereas MMR deficiency was rare, consistent with the predominantly MSS phenotype (Table 3).

**Table 3:** Clinically relevant mutations: gastrointestinal cancer cohort (N=22)

Gene	Pathway	N (%)	Clinical relevance
Tier 1 - Directly targetable			
KRAS	RAS/RAF/MAPK	9 (40.9)	Common GI driver; KRAS alleles guide anti-EGFR use
NRAS	RAS/RAF/MAPK	4 (18.2)	MAPK driver; RAS mutations exclude anti-EGFR in CRC
Tier 2 - Prognostic/resistance			
TP53	Tumor suppressor	16 (72.7)	Associated with aggressive disease and poor prognosis
APC	WNT/beta-catenin	4 (18.2)	Truncal event in colorectal tumorigenesis
SMAD4	TGFβ/SMAD	4 (18.2)	Loss linked to metastatic potential, especially pancreatic/CRC
RB1	Cell cycle	2 (9.1)	Tumor suppressor; adverse biology when lost
KEAP1	Oxidative stress	1 (4.5)	Nrf2 activation; chemoresistance
Tier 3 - Actionable amplifications/cell cycle			
MYC	Cell cycle/proliferation	5 (22.7)	Amplification correlates with aggressive biology
RICTOR	PI3K/AKT/mTOR	2 (9.1)	mTORC2 component; pathway activation
CCND1	Cell cycle	1 (4.5)	G1/S regulator; CDK4/6 targeting interest
Tier 4 - DNA repair/MMR/HRD			
ATM	DNA damage response	2 (9.1)	DDR defect; PARP/ATR inhibitor interest
BRCA1	HRD/DNA repair	1 (4.5)	Hereditary risk; PARP sensitivity
BRCA2	HRD/DNA repair	1 (4.5)	Hereditary risk; PARP sensitivity
BRIP1	HRD/DNA repair	1 (4.5	HRD-associated; hereditary risk
Tier 5 - Pathway modifiers (MAPK/PI3K/WNT)			
PIK3CA	PI3K/AKT/mTOR	2 (9.1)	PI3K activation; targetable in some settings
PTEN	PI3K/AKT/mTOR	2 (9.1)	Loss activates PI3K; resistance to therapies
CTNNB1	WNT/beta-catenin	2 (9.1)	Constitutive WNT signaling
PIK3R1	PI3K/AKT/mTOR	1 (4.5	Regulatory subunit; pathway activation
Tier 6 - Epigenetic/chromatin			
ARID1A	Chromatin remodeling	2 (9.1)	SWI/SNF component; frequent in GI malignancies
ARID1B	Chromatin remodeling	1 (4.5)	SWI/SNF complex; epigenetic
KMT2D	Epigenetic (KMT2D)	1 (4.5)	Histone methyltransferase; widespread effects
BRD4	Chromatin/BET	1 (4.5)	BET family; BET inhibitors in trials
SMARCB1	SWI/SNF	1 (4.5)	Chromatin remodel; loss associated with aggressive disease
Tier 7 - Other/low evidence			
MTAP	Metabolic	3 (13.6)	Co-deleted with CDKN2A/B; PRMT5/MAT2A targeting interest
GATA6	Transcription	2 (9.1)	Amplification in pancreatic/GI cancers; lineage factor

While the pooled gastrointestinal cohort highlights dominant MAPK and WNT pathway alterations with TP53 and SMAD4 loss, clinical implications, such as KRAS status and anti-EGFR relevance, are site-specific, particularly for colorectal cancer. According to National Comprehensive Cancer Network (NCCN) guidelines, KRAS mutations function as a negative predictive biomarker for anti-EGFR therapy. In this cohort, while multiple alterations were theoretically actionable at the molecular level, only a subset met guideline-supported criteria (e.g., KRAS wild-type status for anti-EGFR therapy), with nine of 22 patients (40.9%) harboring KRAS mutations that would preclude cetuximab-based treatment.

**Figure 2:**
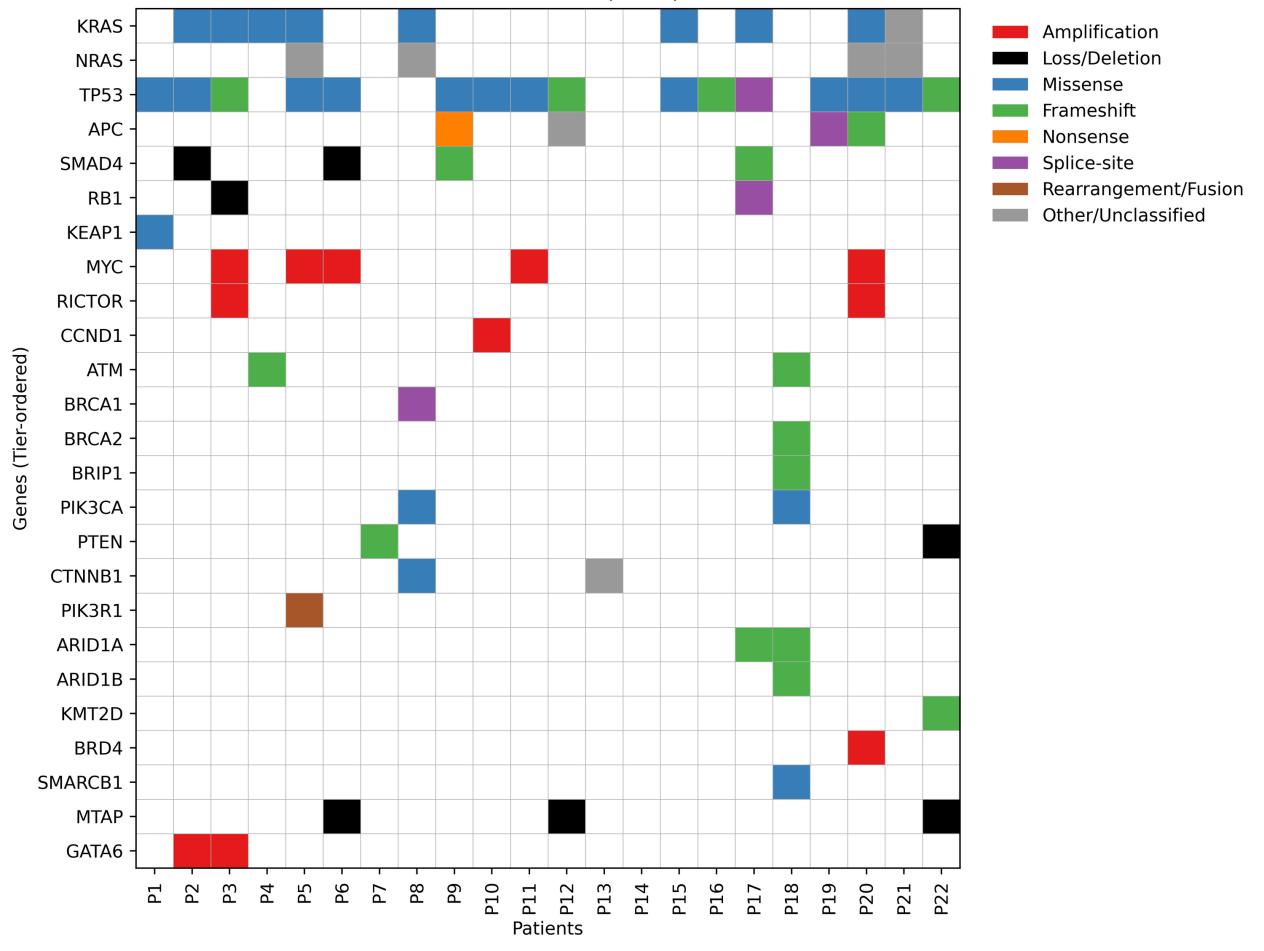
Somatic mutation map of gastrointestinal cancer patients by key oncogenic pathways (N=22)

The absence of MSI-high tumors and the dependence of immunotherapy decisions on additional factors such as PD-L1 expression and disease stage further limit immediate clinical applicability. These numbers are intended to explicitly illustrate the gap between detected genomic alterations and guideline-concordant, clinically actionable treatment options. Overall, gastrointestinal tumors were characterised by MAPK/WNT activation with co-existing PI3K and HRD pathway alterations. The somatic mutation map of gastrointestinal cancer patients across key oncogenic pathways is illustrated in Figure 2.

Within the lung cancer cohort, genomic alterations reflected classical NSCLC biology, including KRAS G12D, RET-rearrangements, and PIK3CA mutations or amplifications. Frequent copy-number gains (SOX2, FGF12, NSD3, BCL2L2) and losses involving CDKN2A/B and RB1 indicated strong cell-cycle dysregulation. Tumor-suppressor gene mutations (TP53, RB1) further supported highly unstable tumor biology (Table 4).

**Table 4:** Clinically relevant mutations: lung cancer cohort (N=18)

Gene	Pathway	N (%)	Clinical relevance
Tier 1 - Directly targetable			
KRAS	RAS/MAPK	4 (22.2)	Common lung driver; G12C and others targetable
EGFR	RTK/RAS	1 (5.6)	Standard actionable driver (EGFR-mutant NSCLC)
BRAF	MAPK	1 (5.6)	BRAF V600E actionable with BRAF/MEK inhibitors
RET	RTK	1 (5.6)	RET fusions actionable (Selpercatinib, Pralsetinib)
Tier 2 - Prognostic/resistance			
TP53	Tumor suppressor	10 (55.6)	Adverse prognosis; genomic instability
CDKN2A/B	Cell cycle	8 (44.4)	Loss linked to CDK dysregulation; poor outcome
RB1	Cell cycle	2 (11.1)	Co-loss with STK11/TP53 defines aggressive subsets
Tier 3 - Actionable amplifications/cell cycle			
MYC	Cell cycle/proliferation	3 (16.7)	High proliferation; poor prognosis
CCNE1	Cell cycle	1 (5.6)	Copy-number gain; chemoresistance; CDK2 targeting interest
SOX2	Lineage TF, squamous	1 (5.6)	Recurrent amp in lung SqCC; oncogenic driver
BCL2L1	Apoptosis	1 (5.6)	Anti-apoptotic; resistance to therapy
BCL2L2	Apoptosis	1 (5.6)	Anti-apoptotic family member
NSD3	Chromatin/8p11 amplicon	1 (5.6)	Part of 8p11 amplicon; co-occurs with FGFR/RTK
ZNF217	Transcription	1 (5.6)	Oncogenic amp; adverse prognosis
AXL	RTK	1 (5.6)	Targetable with AXL inhibitors (trials)
FGF12	FGF/RTK co-amp	1 (5.6)	Cooperating amplification; RTK axis
Tier 4 - DNA repair/MMR (HRD-like)			
BRCA2	HRD/DNA repair	1 (5.6)	PARP inhibitor sensitivity in selected settings
MSH6	MMR	1 (5.6)	MMR defect; potential MSI/ICI relevance if biallelic
PMS2	MMR	1 (5.6)	MMR component; MSI/ICI implications when inactivated
Tier 5 - PI3K/MAPK pathway modifiers			
PIK3CA	PI3K/AKT/mTOR	2 (11.1)	Downstream of RTK; combination-targeting interest
PIK3R1	PI3K/AKT/mTOR	1 (5.6)	Regulatory PI3K subunit; pathway activation
PTEN	PI3K/AKT/mTOR	1 (5.6)	Loss activates PI3K pathway; resistance to some therapies
NF1	RAS/MAPK	1 (5.6)	Loss activates RAS/MAPK; modulates TKI response
NRAS	RAS/MAPK	1 (5.6)	Alternative RAS driver; impacts targeted therapy choices
Tier 6 - Epigenetic/chromatin			
MLL2	Epigenetic (KMT2D)	2 (11.1)	Histone methyltransferase; broad epigenetic effects
ARID1A	Chromatin remodeling	1 (5.6)	SWI/SNF complex; epigenetic vulnerability
BRD4	Chromatin/transcription	1 (5.6)	BET protein; target for BET inhibitors
Tier 7 - Other/low evidence			
MTAP	Metabolic	2 (11.1)	Co-deleted with CDKN2A; potential PRMT5/MAT2A target
GRM3	GPCR	1 (5.6)	Rare; experimental
SRC	Non-receptor TK	1 (5.6)	Potential for SRC inhibitors; low evidence
ARFRP1	Vesicle trafficking	1 (5.6)	Low clinical evidence

**Figure 3:**
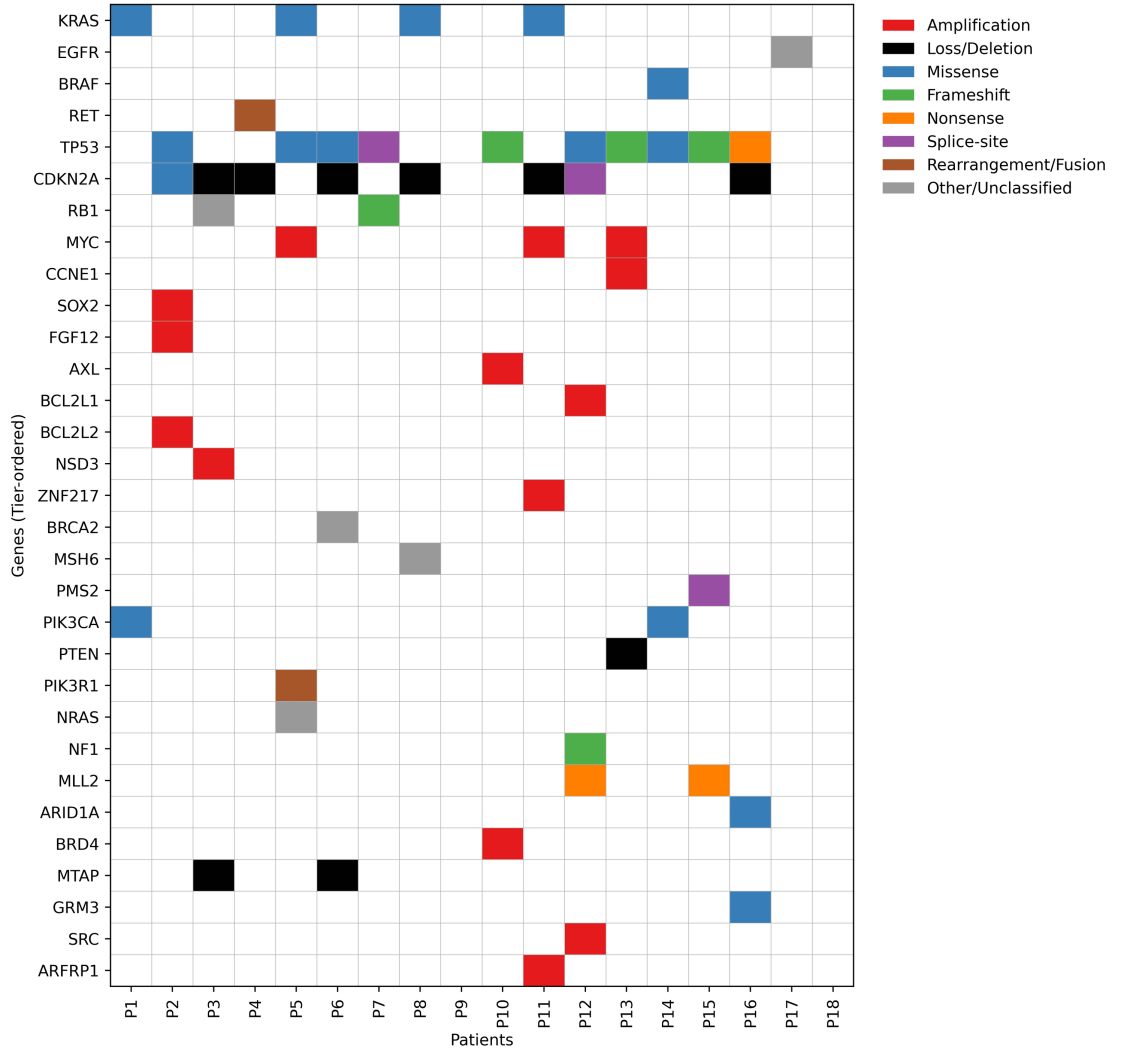
Somatic mutation map of lung cancer patients by key oncogenic pathways (N=18)

Collectively, lung tumors exhibited KRAS/RET-driven MAPK signalling, PI3K activation, and broad tumor-suppressor inactivation. Interestingly, in this cohort, none of the cases were MSI-high, underscoring that immunotherapy decisions should be anchored in programmed death-ligand 1 (PD-L1) status and clinical staging rather than broad genomic findings alone. Although the genomic report indicated a high level of theoretical actionability, the proportion of cases truly actionable as per NCCN guidance was limited, highlighting a clear disconnect between potential drug eligibility and guideline-driven clinical applicability. Figure 3 presents the somatic mutation map of lung cancer patients, highlighting alterations across key oncogenic pathways.

The gynecologic cancers (n=15) showed a landscape dominated by PI3K/AKT/mTOR activation (PIK3CA, PIK3R1/CB, PTEN), frequent TP53 mutations, and amplification-driven events (CCNE1, MYC, FGFR2). MAPK-related genes (KRAS, GNAS, MAP3K1, MAP2K4) and epigenetic regulators (ARID1A, BRD4, ATRX) were recurrently altered. HRD-associated mutations (BRCA1) appeared in a minority (Table 5).

**Table 5:** Clinically relevant mutations: gynecologic cancer cohort (N=15)

Gene	Pathway	N (%)	Clinical relevance
Tier 1 - Directly targetable			
PIK3CA	PI3K/AKT/mTOR	3 (20)	Actionable with PI3K-pathway targeting in some gynec cancers
FGFR2	FGFR/RTK	1 (6.7)	FGFR-targeted agents/clinical trials in endometrial/ovarian cancers
ERBB4	RTK	1 (6.7)	HER family RTK; occasionally targetable
Tier 2 - Prognostic/resistance			
TP53	Tumor suppressor	12 (80)	High-grade serous/aggressive histologies; poor prognosis
PTEN	PI3K/AKT/mTOR	3 (20)	Common in endometrial cancer; pathway activation
RB1	Cell cycle	1 (6.7)	Loss linked to high-grade transformation
KEAP1	Oxidative stress	1 (6.7)	Nrf2 activation; chemoresistance
Tier 3 - Actionable amplifications/cell cycle			
CCNE1	Cell cycle	2 (13.3)	Associated with platinum resistance in ovarian cancer
MYC	Cell cycle/proliferation	1 (6.7)	High proliferative index; adverse prognosis
CCND3	Cell cycle	1 (6.7)	G1/S control; CDK4/6 targeting interest
Tier 4 - HRD/DNA Repair/MMR			
BRCA1	HRD/DNA repair	2 (13.3)	PARP inhibitor sensitivity; hereditary breast/ovarian
Tier 5 - PI3K/MAPK modifiers			
KRAS	MAPK/RAS	4 (26.7)	KRAS mutations in endometrioid/low-grade serous tumors
PIK3R1	PI3K/AKT/mTOR	1 (6.7)	Regulatory PI3K subunit; pathway activation
PIK3CB	PI3K/AKT/mTOR	1 (6.7)	PI3K isoform; pathway signalling
NF1	MAPK/RAS	1 (6.7)	Loss activates RAS/MAPK; endocrine/targeted resistance
GNAS	MAPK/G-protein	1 (6.7)	Activating GNAS mutations in mucinous/rare tumors
MAP3K1	MAPK	1 (6.7)	MAPK pathway modulation
MAP2K4	MAPK	1 (6.7)	Stress-activated MAPK node
MTOR	PI3K/AKT/mTOR	1 (6.7)	Downstream mTOR; pathway activation
Tier 6 - Epigenetic/chromatin			
ARID1A	Chromatin remodeling	4 (26.7)	Common in ovarian clear cell/endometrioid; therapeutic vulnerability
MLL2	Epigenetic (KMT2D)	2 (13.3)	Histone methyltransferase; epigenetic dysregulation
BRD4	Chromatin/BET	1 (6.7)	BET family; BET inhibitors in trials
ATRX	Chromatin	1 (6.7)	Chromatin remodel; ALT phenotype
Tier 7 - Other/low evidence			
NOTCH3	NOTCH signalling	1 (6.7)	Associated with chemoresistance and poor outcome in ovarian CA
SGK1	Serine/threonine kinase	1 (6.7)	Downstream of PI3K; emerging target
SPOP	Ubiquitin signalling	1 (6.7)	Alterations in endometrial cancers; functional impact
MTAP	Metabolic	1 (6.7)	Co-deletion with CDKN2A; PRMT5-targeting interest
CDKN2A/B	Cell cycle	1 (6.7)	Loss leads to CDK4/6 activation

**Figure 4:**
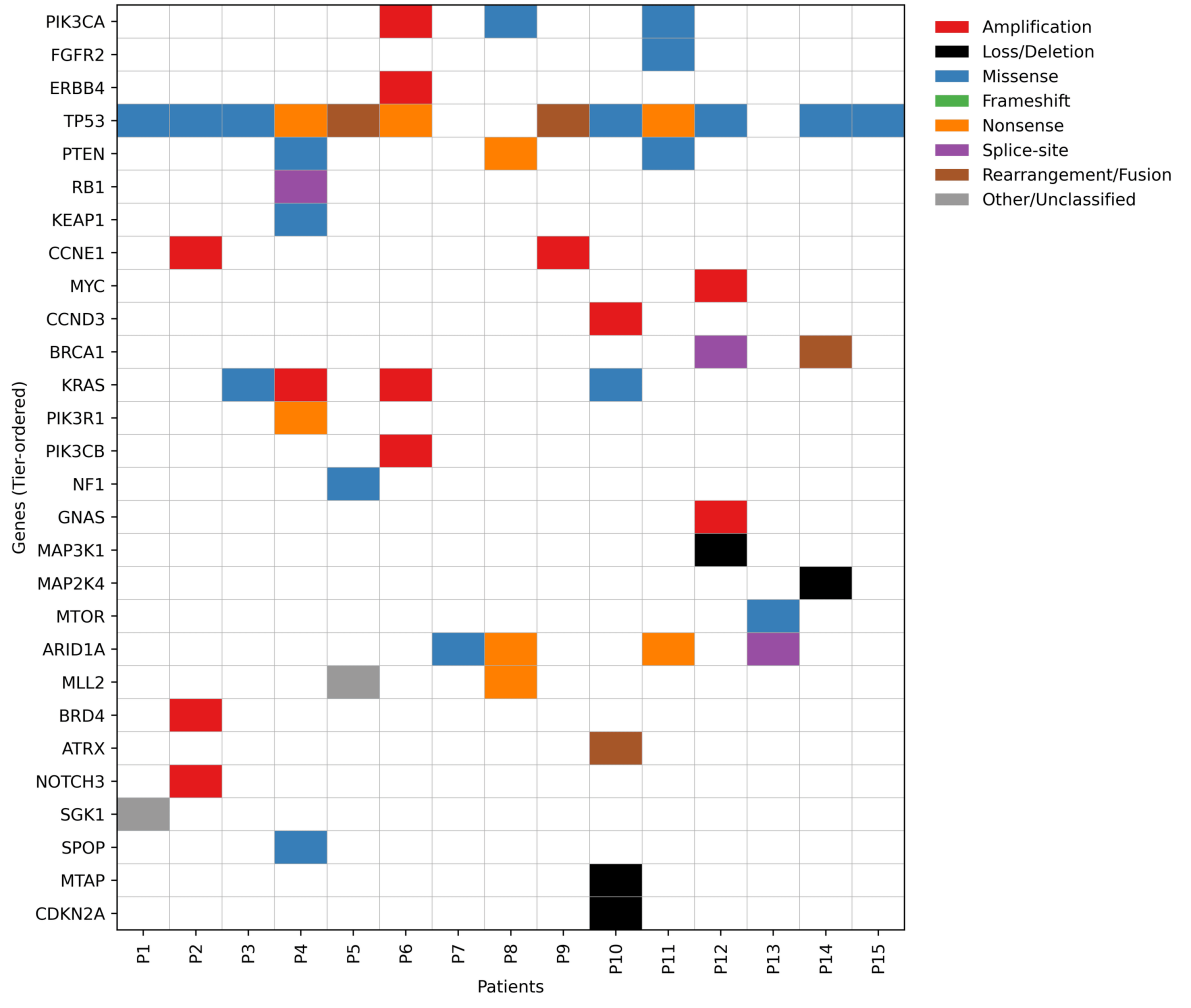
Somatic mutation map of gynecologic cancer patients by key oncogenic pathways (N=15)

These findings reflect pathway convergence across PI3K, MAPK, and cell-cycle signalling. The somatic mutation map of gynecologic cancer patients across key oncogenic pathways is illustrated in Figure 4.

In Head & Neck cancers (n=9), TMB was uniformly low, and all cases were MSS. Key alterations included MAP2K1, EGFR amplification, FGF12 gain, and pervasive TP53 disruption, alongside copy-number gains in CCND1, PRKCI, and TERC. These findings indicate dominant MAPK/RTK signalling with strong cell-cycle amplification. The GBM cohort displayed low TMB and classic high-grade glioma drivers: EGFR amplification, PTEN loss, CDKN2A/B deletion, TERT promoter mutations, and, in some cases, IDH1 R132H with CIC/FUBP1/BCOR co-alterations. Additional events included FGFR3-TACC3 fusion, CTNNB1 mutation, and MTAP loss, reflecting RTK/PI3K activation, p53/PTEN pathway disruption, and telomerase activation.

The prostate cancer cohort showed variable TMB (4-53 muts/Mb) with one MSI-high case. The identification of MSI-high in one patient represents a rare but clinically significant finding. This result may be attributable to the observed MSH2 complex rearrangement and confers eligibility for immunotherapy, making it an important actionable event within this cohort. Frequent DNA damage response (DDR)-related alterations (BRCA2, MSH2, ATM, FANCG) were observed alongside WNT/MAPK-associated variants (CTNNB1, NF1, NOTCH2) and AR pathway mutations. Overall, tumors displayed DDR deficiency, AR-pathway disruption, and MAPK/WNT activation, indicating clear therapeutic implications. The liver cancer samples exhibited low TMB and MSS biology, with recurrent KRAS mutations, TP53 loss, MDM2 amplification, and deletion of RB1, CDKN2A/B, and chromatin regulators (SMARCA4, DNMT3A). These changes reflect aggressive hepatobiliary tumor biology dominated by MAPK signalling and p53/TERT-pathway dysregulation.

The urology cohort showed low-TMB, MSS tumors. Kidney cancers harboured VHL splice variants and PBRM1 loss with additional alterations in NF1, KDM5C, CREBBP, and TET2. The bladder tumor demonstrated amplifications in MYC, BCL2L1, MCL1, VEGFA, and mutations in AKT2, RB1, TP53, and TERT promoter, indicating aggressive urothelial carcinoma biology with MAPK/PI3K activation and cell-cycle deregulation. Finally, the sarcoma samples were characterised by extremely low TMB and MSS status. One case showed no pathogenic alterations, while the fusion-driven tumor exhibited a classic FUS-FLI1 fusion with MLL2 truncation, consistent with Ewing sarcoma biology and minimal background mutational noise.

Collectively, the findings highlight shared actionable pathways and distinct molecular vulnerabilities across diverse solid malignancies. Overall, the F1CDx report recommended FDA-approved therapies relevant to the patient’s own tumor type in 48 cases (41.7%), while suggesting FDA-approved therapies from other tumor types in 58 cases (50.4%). The majority of cases in our cohort showed low TMB and were MSS, suggesting limited mutational load and a genomic profile less likely to be associated with immunotherapy responsiveness.

## Discussion

Certain cancers contain oncogenic driver mutations that predominantly drive tumor growth, creating opportunities for targeted therapies [19]. There has been a shift toward tumor-agnostic therapies that target genetic alterations irrespective of tumor origin, emphasizing the need to identify actionable mutations as targeted options expand [13]. Biomarker-matched therapies have been shown to improve survival across several malignancies, and the number of druggable genomic alterations continues to grow [20]. Over the past decade, the adoption of NGS has transformed cancer diagnosis and treatment planning [20]. Its high-throughput and cost-effective nature makes it central to tumor detection, prognostication, and therapeutic decision-making [7, 20], while also enabling broader multi-omics research [21]. NGS testing has been shown to influence response rates and progression-free survival, and platforms such as Foundation Medicine can detect a wide range of clinically actionable mutations [3]. In this context, the present study evaluates the mutational landscape of a diverse Indian cohort with advanced cancers who underwent comprehensive genomic profiling.

The demographic and clinical characteristics of our cohort align closely with those reported in previous studies [3,5,13,22]. In line with previous reports, our cohort showed a female predominance (67.8%), a pattern also observed in other NGS-based mutational profiling studies [3,22]. Similarly, the median age in our cohort aligns with prior studies reporting a median age of about 56 years (range: 19-84) among patients undergoing NGS-based Foundation testing [3]. In our series, breast cancer was the most common DMG, followed by gastrointestinal malignancies, thoracic/lung cancers, and gynecologic cancers, while head and neck cancers, GBM/primary brain tumors, prostate and other urologic cancers, liver cancer, and sarcomas were less frequently represented.

This distribution contrasts with previously published studies. For instance, one report identified gastrointestinal cancers (33.4%) as the predominant DMG, followed by genitourinary (15.5%), gynecologic (11.4%), breast (10.6%), central nervous system (CNS) (8.6%), and sarcoma (6.9%) tumors, with lower frequencies of head and neck, melanoma, neuroendocrine, lung, mesothelioma, and thymoma cancers [5]. The same study also reported differing frequencies of individual tumor types, with breast (11%), colorectal (10%), CNS (9%), pancreatic (8%), sarcoma (7%), cholangiocarcinoma (6%), and ovarian cancers (6%) being the most common [5]. Another study similarly documented lung (23%), colorectal (19%), breast (13%), and head and neck cancers (7%) as the most prevalent DMGs [13].

In our study, breast tumors predominantly demonstrated low to intermediate TMB and were largely MSS. This is consistent with earlier work showing a median TMB of 4 mut/Mb in early-stage triple-negative breast cancer (TNBC) and 2.6 mut/Mb across broader breast cancer cohorts [23]. Similarly, comprehensive genomic profiling using F1CDx has reported a median TMB of 4 (range 0-16), further supporting our observations [24]. The genomic architecture of breast cancer in our cohort was characterized mainly by TP53 mutations (62.9%), substantial activation of the PI3K/AKT pathway (PIK3CA ~37%; PTEN ~14%), and recurrent 8p11-12/11q13 amplifications involving FGFR1, NSD3, ZNF703, CCND1, and MYC. Additional alterations included ERBB2 (8.6%), CDH1 (8.6%), and HRD-associated genes such as BRCA1 and RAD21.

These findings broadly align with previously published genomic profiling studies. A large F1CDx-based series demonstrated a higher TP53 mutation frequency (95%), accompanied by recurrent alterations in MYC (23%), RAD21 (22%), PIK3CA (19%), BRCA1 (17%), PTEN (15%), FGFR1 (12%), NSD3 (12%), ZNF703 (11%), NF1 (11%), PIK3R1 (10%), RB1 (9%), and BRCA2 (9%) [24]. Although their TP53 rate exceeded ours, likely reflecting differences in patient selection and tumor subtype distribution, the overall spectrum of co-occurring mutations is comparable to our study. Prior analyses also show that basal-like and TNBC tumors harbor high rates of TP53 (≈80%) and PIK3CA mutations, supporting the high proportion of these alterations observed in our cohort [25].

Similarly, subtype-specific NGS studies have reported recurrent involvement of PIK3CA, ERBB2, ESR1, FGFR1, and PTEN across TNBC, HR+, and ERBB2+ disease, paralleling the mutational pattern identified in our series [26]. Pathway-level comparisons further corroborate our results. Previous work identified the RTK/RAS pathway as the most frequently altered signaling cascade (81.4%), predominantly through alterations in ERBB2 (33.3%), FGFR1 (21.2%), and NF1 (15.2%) [26]. In our cohort as well, we observed recurrent ERBB2 amplification and FGFR1 involvement within the 8p11-12/11q13 amplicons, mirroring these pathway disruptions. Likewise, alterations in the PI3K/mTOR/AKT pathway, reported previously in 65.8% of breast cancers, were strongly represented in our data, with PIK3CA and PTEN being among the most commonly altered genes. Prior studies have also described changes in the cell-cycle, DNA-repair, p53, and MYC pathways [26], all of which were reflected to varying degrees within our cohort, particularly through CCND1 amplification, BRCA1 involvement, and recurrent MYC and TP53 alterations.

Our findings also resonate with earlier reports highlighting TP53 as a central driver of breast carcinogenesis, particularly in TNBC, where mutation rates approach 60% [27-28]. Beyond single-gene alterations, previous studies have identified recurrent amplifications involving MYC (8q24), FGFR1 (8p11.23), and CCND1 (11q13), amplifications that we likewise observed. These regions contribute to distinct genomic subclusters, often associated with immune-evasive phenotypes and proliferative signaling [24]. Moreover, the presence of 11q13 and 8p11.23 amplicons, previously shown to define specific TNBC clusters [24], was also evident in our cohort. The 11q13 amplicon, in particular, which includes CCND1 and several FGF genes, has been linked to therapeutic resistance and aggressive tumor evolution [29-30], underscoring its clinical significance in both prior literature and our dataset. Overall, the genomic landscape observed in our breast cancer cohort, including TMB range, dominant driver mutations, pathway disruptions, and recurrent amplicons, is highly concordant with previously published studies, reinforcing the biological consistency of breast cancer subtypes across populations while contributing additional insights from an Indian clinical context.

In our study, the gastrointestinal cancer cohort exhibited a mutation profile dominated by MAPK-pathway activation (KRAS, NRAS) and WNT-pathway dysregulation (APC, CTNNB1), along with widespread TP53 and SMAD4 loss. Additional alterations involved PI3K-pathway components (PIK3CA, PIK3R1, PTEN). A subset of tumors also demonstrated HRD-associated mutations (BRCA1/2, ATM, BRIP1), whereas MMR deficiency was uncommon, consistent with the predominantly MSS phenotype observed. These findings align with prior genomic profiling studies. In a prospective analysis of 116 predominantly locally advanced or metastatic gastric cancers, 78% of cases harbored at least one clinically relevant alteration. The most frequent mutations involved TP53 (50%), ARID1A (24%), KRAS (16%), CDH1 (15%), CDKN2A (14%), CCND1 (9.5%), ERBB2 (8.5%), PIK3CA (8.6%), MLL2 (6.9%), FGFR2 (6.0%), and MET (6.0%).

RTK alterations were present in 20.6% of cases, primarily ERBB2, FGFR2, and MET amplifications, with ERBB2 alterations evenly divided between amplifications and base substitutions. Rare BRAF mutations (2.6%) were also reported [31]. Similarly, another study showed that in primary gastrointestinal tumors, actionable genomic alterations were detected in 67% of patients, most commonly PIK3CA short variants (33%), as well as ERBB2 (28%) and EGFR (17%) amplifications [32]. Together, these studies corroborate our findings, particularly the prominence of MAPK, WNT, RTK, and PI3K signaling alterations and the relative rarity of MMR deficiency in gastrointestinal malignancies. Furthermore, a recent review of diagnostic innovations in pancreatic cancer has advocated the potential role of comprehensive genomic profiling in identifying actionable molecular alterations that may inform individualized treatment strategies in this challenging disease setting [33].

In this study, the lung cancer cohort demonstrated a range of clinically relevant genomic alterations, including KRAS G12D, RET rearrangements, PIK3CA mutations or amplifications, and tumor-suppressor gene alterations involving TP53 and RB1. Evidence of cell-cycle dysregulation was also seen through frequent copy-number gains (SOX2, FGF12, NSD3, BCL2L2) and losses (CDKN2A/B, RB1). These findings closely parallel those from a prior F1CDx-based analysis, which identified the most frequent alterations in lung cancer as KRAS (12%), followed by EGFR (11%), MET (4%), ERBB2 (4%), ALK (2%), BRAF (1%), RET (0.7%), ROS1 (0.6%), and NTRK1/2/3 (0.2%) [34]. Notably, none of the lung tumors in our cohort were MSI-high, suggesting that immunotherapy decisions in this population should rely primarily on PD-L1 expression and clinical staging rather than MSI status. This contrasts with a previous large series of NSCLC samples assessed using the F1CDx panel, in which TMB ≥10 mut/Mb was observed in 34% of cases and MSI-high status in 4% [34].

PD-L1 testing remains a critical companion diagnostic, as reflected in FDA approvals and NCCN guidelines for advanced NSCLC [34]. Furthermore, consensus recommendations from the Spanish Society of Medical Oncology (SEOM) and the Spanish Society of Pathology (SEAP) emphasize the need to detect key actionable biomarkers in non-squamous NSCLC, including EGFR, BRAFV600E, KRASG12C, and MET exon 14 skipping mutations, along with ALK, ROS1, RET, and NTRK gene fusions, and PD-L1 expression [35]. Our mutational findings are consistent with these recommended testing priorities and underscore the clinical relevance of comprehensive genomic profiling in lung cancer management.

In our gynecologic cancer cohort, the mutational landscape was characterized by prominent PI3K/AKT/mTOR pathway activation (PIK3CA, PIK3R1/CB, PTEN), frequent TP53 mutations, multiple amplification-driven events (CCNE1, MYC, FGFR2), recurrent MAPK-pathway alterations (KRAS, GNAS, MAP3K1, MAP2K4), and disruptions in epigenetic regulators such as ARID1A, BRD4, and ATRX, while HRD-associated mutations (BRCA1) appeared only in a minority of cases. These findings are broadly aligned with earlier genomic studies; for example, Foundation Medicine-based profiling identified TP53 (75%), KRAS (21%), PIK3CA (21%), PPP2R1A (11%), and CCNE1 (10%) as the most frequently altered genes in gynecologic cancers [36]. Subtype-specific patterns have also been described, with endometrioid tumors showing alterations in KRAS, CDKN2A, CCND1, CTNNB1, PIK3CA, and PTEN, and low-grade serous carcinomas demonstrating KRAS and NRAS mutations but lacking TP53 alterations [36].

Further pathway-level analyses from other studies support our observations, highlighting recurrent alterations across six major functional groups, including cell-cycle regulation, DDR, p53 signaling, PI3K-AKT-mTOR, RAS-RAF-MEK/JNK, and RTK pathways [34]. Moreover, another study utilizing the OmniSeq comprehensive test (90% of cases) and the Foundation Medicine panel (10%) reported a broad spectrum of genetic alterations across common gynecologic cancer subtypes. In high-grade serous carcinoma, frequent mutations included TP53 (56.7%), BRCA1 (26.1%), BRCA2 (17.2%), NF1 (16.6%), and PIK3CA (12.7%). Endometrioid carcinomas commonly harbored alterations in PIK3CA (51%), PTEN (43.1%), ATM (23.5%), KRAS (17.6%), and CTNNB1 (17.6%). Squamous cell carcinomas showed frequent PIK3CA (35.3%), FBXW7 (17.6%), NFE2L2 (17.6%), PD-L1 (17.6%), and BIRC3 (11.6%) alterations.

Carcinosarcomas demonstrated recurrent TP53 (56.2%), PIK3CA (50%), APC (25%), ATM (25%), and PTEN (25%) mutations. In clear cell carcinoma, PIK3CA (36.4%), BRCA1 (27.3%), BRCA2 (18.2%), JAK3 (9.1%), and NOTCH1 (9.1%) were commonly altered, whereas low-grade serous carcinomas frequently exhibited APC (25%), BRCA2 (25%), KRAS (25%), NRAS (25%), and CDKN2A (25%) alterations [35]. Notably, ARID1A mutations were also observed in this cohort, including one case of clear cell carcinoma and three cases of high-grade serous carcinoma, underscoring its recognized role in the pathogenesis of several gynecologic cancer subtypes [35]. Collectively, these results demonstrate strong concordance between our findings and previously published genomic profiles, reinforcing the central roles of PI3K-pathway activation, TP53 disruption, MAPK signaling, and amplification-driven oncogenic events across gynecologic malignancies.

In this study, we also evaluated genomic alterations in several less frequent cohorts. In the prostate cancer cohort, the alteration spectrum closely mirrored previously published findings, which identified TP53 mutation or loss (39%), TMPRSS2-ERG fusion (34%), PTEN loss (31%), and AR amplification (20%) as the most common events [37]. Additional analyses from earlier studies have reported clinically relevant mutations such as ATM and BRCA2 alterations, PALB2 and FANCA loss (Level 3A), PD-L1 and CDK4 amplification and PMS2 mutations (Level 3B), and CDK12 and CDKN2A/B loss (Level 4) in prostate tumors [37], supporting the diversity of DNA repair and cell-cycle defects observed in our cohort. In the liver cancer cohort, tumors displayed low TMB, MSS biology, and a mutational profile dominated by MAPK pathway activation and p53/TERT-axis dysregulation, consistent with aggressive hepatobiliary tumor biology. Prior studies have similarly identified TP53 (33.3%) and CTNNB1 (33.3%) as the most frequent alterations in hepatocellular carcinoma, along with a median TMB of 4 (range 0-20), rare TMB-high cases (TMB ≥ 10 muts/mb), and absence of MSI-high tumors [38], corroborating our findings.

Other less common tumor types in our study, including head and neck cancers, urologic malignancies, sarcomas, and GBM, also exhibited low TMB and predominantly MSS status. In head and neck cancers, key alterations involved MAP2K1, EGFR amplification, FGF12 gain, pervasive TP53 disruption, and copy-number gains in CCND1, PRKCI, and TERC, consistent with established oncogenic drivers in this group. The GBM cohort showed the expected pattern of RTK/PI3K pathway activation, p53/PTEN pathway loss, and telomerase activation, aligning with canonical molecular features of high-grade gliomas. In the bladder cancer sample, alterations reflected aggressive urothelial carcinoma biology characterized by MAPK and PI3K pathway activation and pronounced cell-cycle deregulation.

Strengths and limitations

This study has several notable strengths. It represents one of the few real-world evaluations of comprehensive genomic profiling using an FDA-approved NGS panel (F1CDx) in an Indian oncology setting, spanning a wide range of solid tumors over an extended time period. The use of a standardized data-extraction pipeline, patient-level alteration matrix, and pathway-oriented analysis enabled consistent characterization of somatic events across diverse malignancies and facilitated meaningful comparison with international datasets. Importantly, the study provides granular insights into the frequency and clinical actionability of genomic alterations in a low- and middle-income country context, where access to precision oncology remains constrained.

However, certain limitations must be acknowledged. The retrospective, single-center design and relatively modest overall sample size, particularly within individual tumor subgroups, may limit the generalizability of the findings. Referral and selection bias are likely, as only patients who could access or were referred for NGS testing were included. The hierarchical selection of a single highest-impact alteration per gene may under-represent clinically relevant co-alterations, including resistance mechanisms or dual hits, which are better appreciated at the individual patient level. Detailed data on downstream treatment decisions, treatment uptake based on NGS results, and long-term clinical outcomes (e.g., response rates, progression-free or overall survival) were not systematically available, precluding a robust assessment of the real-world impact of genomically matched therapies.

## Conclusions

This retrospective, real-world analysis shows that comprehensive genomic profiling with the F1CDx assay is feasible and clinically informative in an Indian oncology setting. Across 115 patients, recurrent alterations were identified in key oncogenic pathways, including TP53, PI3K/AKT/mTOR, MAPK, RTK, WNT, DNA-repair, and cell-cycle regulation, with mutational patterns largely consistent with global datasets. Although genomic actionability in this study largely reflects report-level or biological findings, guideline-concordant treatment options were more limited, owing to the predominance of MSS/low-TMB tumors and restricted access to targeted therapies. Notably, rare but clinically meaningful findings such as IDH1 mutations in atypical tumors and MSI-high prostate cancer highlight the value of broad-panel NGS in detecting actionable outliers that routine testing may miss. As NGS becomes more integrated into oncology practice, it has the potential to expand access to targeted and tumor-agnostic therapies. Prospective studies with treatment and outcome follow-up are needed to determine whether comprehensive genomic profiling translates into improved clinical benefit over traditional site-specific molecular testing.
